# Fibrocalculous pancreatic diabetes: a case report

**DOI:** 10.1186/s13104-015-1142-8

**Published:** 2015-04-30

**Authors:** Dissanayake Mudiyanselage Priyantha Udaya Kumara Ralapanawa, Kushalee Poornima Jayawickreme, Ekanayake Mudiyanselage Madhushanka Ekanayake

**Affiliations:** Consultant Physician & Senior Lecturer, Department of Medicine, University of Peradeniya, Peradeniya, Sri Lanka; Temporary Lecturer and Research Assistant, Department of Medicine, University of Peradeniya, Peradeniya, Sri Lanka

**Keywords:** Diabetes Mellitus, Chronic pancreatitis, Fibrocalculous pancreatopathy, Tropical pancreatitis, Sri Lanka

## Abstract

**Background:**

Diabetes is now becoming a major cause of morbidity and mortality in both developing and developed countries. Even though type 1 and type 2 are the commonest, diabetes mellitus due to secondary causes have been identified. Fibrocalculous Pancreatic Diabetes is a unique entity wherein pancreatic calcification and chronic inflammation lead to exocrine and endocrine failure of the pancreas. This form of non-alcoholic pancreatopathy is exclusively seen among the young, with a male preponderance and commonly in tropical countries where malnutrition and poverty go hand in hand. Whereas, interestingly this case has a late presentation in a female, unlike in other reported cases. For the best of our knowledge this is the first such documented case reported in Sri Lanka.

**Case presentation:**

A 57 year old non-alcoholic Sinhalese female from Sri Lanka, presented with a history of chronic pancreatitis of nine years duration, after which she had developed severe Insulin Dependent Diabetes Mellitus. Imaging of the abdomen showed typical pancreatic calcifications, and this presentation accords with the criteria for Fibrocalculous pancreatic diabetes.

**Conclusion:**

This case report demonstrates a rare form of secondary diabetes in a middle aged female, without a childhood history of abdominal pain suggestive of pancreatitis, indicating late onset disease. Therefore a high index of suspicion is necessary even though the diagnostic criteria indicates the presence of childhood onset of disease.

## Background

Fibrocalculous pancreatic diabetes mellitus (FCDP) is a unique form of diabetes mellitus, which is secondary to chronic calcification of the pancreas in young non-alcoholic people and exclusively found in tropical countries [1]. The hall-marks of the disease are the occurrence of abdominal pain since childhood and pancreatic calculi associated with dilatation of the pancreatic duct and fibrosis of the gland in adolescence [2]. Various terminologies have been proposed for this form of diabetes, including Pancreatic Diabetes, Pancreatogenous Diabetes, and Tropical Pancreatic Diabetes. Recently the term Fibrocalculus Pancreatic Diabetes was introduced for this form of diabetes by the World Health Organization (WHO) Study Group Report on Diabetes [2,3].

There is only one reported population based study on the prevalence of tropical chronic pancreatitis. Balaji made a systematic survey of 6079 families of a district in Kerala. A population of 28,507 was studied and 1 in 1020 subjects had Chronic calcific pancreatitis (0.09%) [4].

## Case presentation

A 57 year old previously well Sinhalese Sri Lankan female who was referred to the Diabetic clinic in a Teaching Hospital, had a past history of intermittent epigastric discomfort for the past nine years which was initially attributed to and treated for upper gastrointestinal symptoms. The abdominal pain was intermittent, burning in nature and radiated to the back, associated with steatorrhea and subjective weight loss. She had no past history of consumption of alcohol or cassava, gall stone disease or hepatitis, and no family history of pancreatic disease or diabetes mellitus.

Her initial blood sugar values were in normal range, but on routine follow up she was detected with poor glycemic control with an elevated fasting plasma sugar level of 128mgdl^-1^ and HbA1c of 14.6% and, was started on oral hypoglycemics. Later she was changed on to biphasic insulin four years ago due to poor glycemic control, and her current insulin regimen is 10U mane and 5U vesper. Interestingly she does not have a history of clinical presentation similar to ketoacidiosis even with poor glycemic control. She has no osmotic symptoms, and no clinical evidence of macrovascular or microvascular complications of diabetes, though she has not had any nephrology or ophthalmology referral so far. Until now she was managed for chronic pancreatitis medically by 30,000 IU of pancreatic enzyme supplements daily, along with other micronutrients, and surgically by annual Endoscopic Retrograde Cholangio Pancreatography (ERCP) and stenting for which she has a good response to pain.

On examination she was emaciated, with a Body Mass Index (BMI) of 18.1kgm^-2^, mildly pale, not icteric, no peripheral stigmata of chronic liver disease, and no features of hemochromatosis.

**Figure 1:**
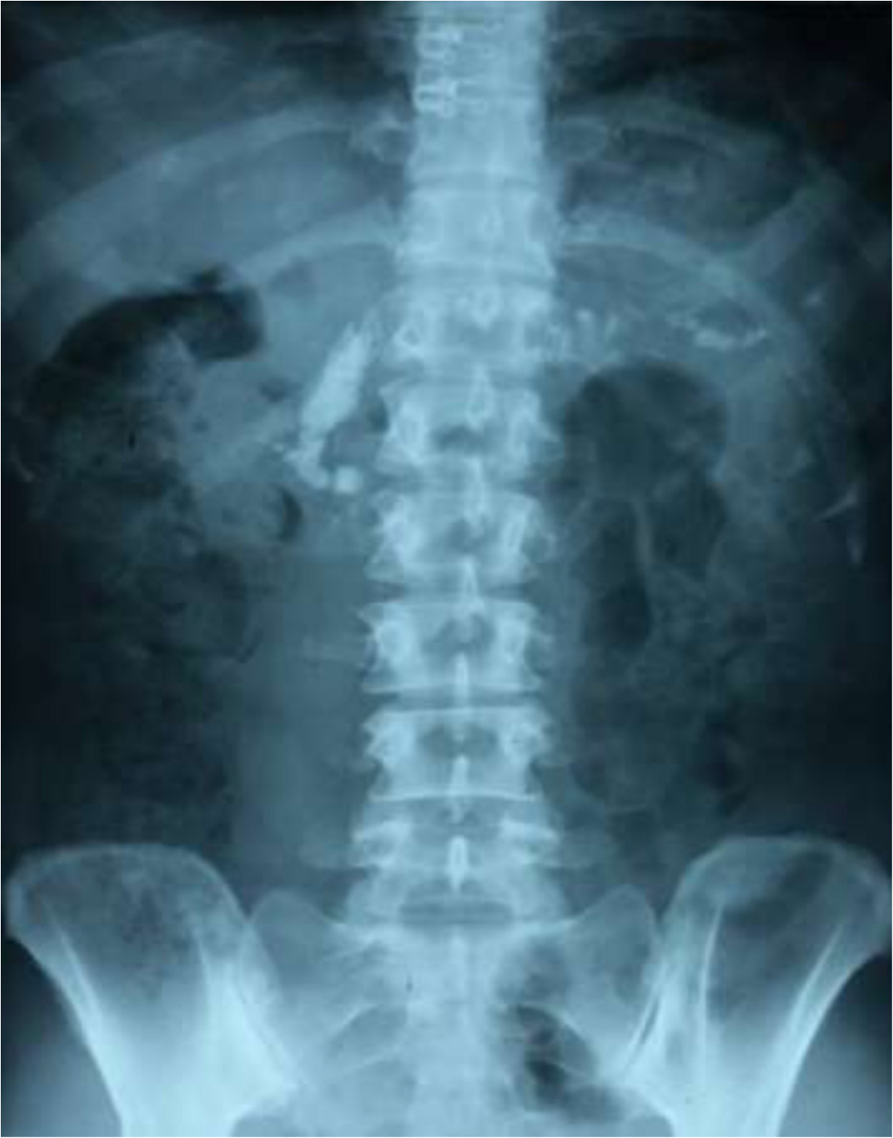
X-ray abdomen showing generalized pancreatic calcification involving head, body and tail of the pancreas, more significantly to the right of the twelfth thoracic and first lumbar vertebrae.

**Figure 2:**
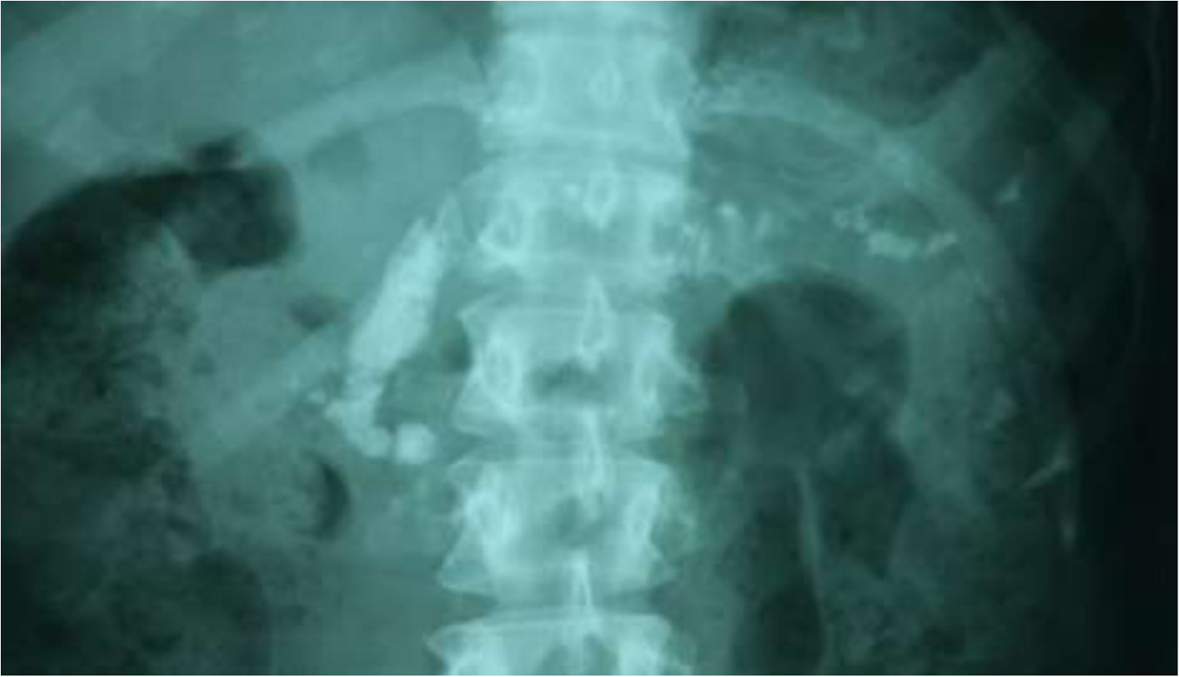
Magnified view of the X-ray abdomen showing generalized pancreatic calcification.

On X-ray abdomen the pancreas was diffusely calcified, involving the head, body and tail, and changes similar to chronic pancreatitis [Figures 1 and 2]. Ultrasonography (USS) and Computed Tomography (CT) scanning of the abdomen showed pancreatic atrophy and the typical “Bag of stones” appearance.

Full Blood count revealed hypochromic microcytic anaemia with a Haemoglobin of 11.5 g/dl, and iron deficiency anaemia was confirmed by iron studies with a Transferrin saturation of 13% (20-50%). Alkaline Phosphate was marginally elevated (218 IU/l), and serum protein was low. Thyroid Function tests were normal with a Thyroid Stimulatimg Hormone (TSH) of 3.53micIU/ml. Lipid profile showed low levels. Urinalysis showed marginal microalbuminuria with a urine albumin:creatinine ratio of 30.39.

Carbohydrate Antigen (CA19-9) was 13U/ml. C-Peptide level was 0.5 nmol/l (normal 0.5- 1.15). Based on these findings, the diagnosis of FCDP was made. Treatment was based on glycaemic control, management of pain, exocrine and endocrine functions of the pancreas. On follow up the patient showed clinical improvement with a BMI of 19.7kgm^-2^, good glycaemic control, and is currently free of pain.

## Discussion

FCPD is a unique form of diabetes mellitus secondary to pancreatic calcification in non-alcoholics, predominantly seen in the young [1]. Diagnosis of diabetes mellitus in FCPD is usually between the ages 10-40, with many having a past history of severe epigastric pain characterized by remissions and exacerbations [5]. Juvenile onset FCPD has been described in cases reported in Africa [6,7] and Asia [8,9]. Unlike classical cases, this patient presented with features of FCPD at the age of 49 years, which is of late onset, with no childhood history of such. A previous case report shows two cases of FCPD in elderly which is very rare [10] similar to this case.

The aetio-pathogenesis of FCPD is thought to be multi factorial, including; malnutrition, toxic effects of cyanide derived from frequent cassava consumption, familial aggregation; being about 10%, genetic factors; which is proposed as the main etiology, and increased oxidant stress from vitamin C and A deficiencies [5]. Current evidence confirms a link between the serine protease inhibitor, Kazal type 1 (SPINK 1) gene and Tropical calcific pancreatitis [11,12]. It is a vital protease inhibitor that prevents unregulated or inappropriate activation of the pancreatic enzyme cascade by inhibiting trypsin activity [13]. This eventually causes recurrent pancreatitis.

The classical triad of FCPD is abdominal pain, steatorrhoea, and diabetes and is associated with overt protein calorie malnutrition. Despite excellent clinical descriptions of the disease, there are no definite criteria for the diagnosis of FCPD. Mohan *et al* [14] have proposed the following criteria for the diagnosis of FCPD, based on their own studies and extensive review of the literature.

### Diagnostic criteria for fibro-calculous pancreatic diabetes (Ref. [15])

1. Occurrence in a “tropical” country.
2. Diabetes by WHO Study Group [1] criteria.
3. Evidence of chronic pancreatitis: pancreatic calculi on X-ray or at least three of the following:
  - Abnormal pancreatic morphology by ultrasonography
  - Chronic abdominal pain since childhood
  - Steatorrhoea
  - Abnormal pancreatic function test.
4. Absence of other causes of chronic pancreatitis. i.e. Alcoholism, hepatobiliary disease or primary hyperparathyroidism etc.

Features like clinical malnutrition, young age at onset and absence of ketosis are commonly associated features, but are not diagnostic criteria by themselves [5]. Though this patient has no chronic abdominal pain since childhood all other criteria for the diagnosis of FCPD are fulfilled.

The classic plain radiographic feature of FCPD is the presence of coarse, well-defined, and dense pancreatic calcifications [15,16]. The calculi are mostly situated to the right of the first or second lumbar vertebrae, but may rarely overlap the spine [5], like in this case. Ultrasound scan and CT scans are useful tools in the diagnosis of FCPD. CT studies done by Yajnik showed that pancreatic mass was preserved in early stages with swelling of parenchyma, followed by varying degrees of atrophy with progress of disease, and finally being replaced by the “Bag of stones” appearance in extreme cases [17] such as in this case.

Malnutrition and low BMI associated with FCPD is multifactorial. This case reports a significant loss of weight, with a current BMI of 18.1 kg/m^2^. A 7 year follow-up study done in Pune [18] showed a low BMI in 72% of these Insulin Dependent Diabetes Mellitus (IDDM) patients implying that diabetes-related malnutrition is a significant factor, although exocrine pancreatic deficiency would also add to the problem. In addition, malnutrition in early life is associated with beta-cell dysfunction and glucose intolerance in later life [19-21]. Ketosis resistance of these patients, also seen in this case, helps distinguish them from the usually ketosis-prone IDDM patients; which is possibly multifactorial in origin [22]. The hypotheseses to explain the ketosis resistance in Malnutrition related Diabetes are summarized by Yajnik [17] as:

1. Residual B-cell function adequate to prevent ketosis
2. Concomitant destruction of A-cells and thus loss of glucagon, a major ketogenic hormone
3. Subcutaneous fat loss and therefore, reduced supply of Non Esterified fatty acids (NEFA) - the “fuel” for ketogenesis
4. Resistance of subcutaneous adipose tissue lipolysis to adrenaline
5. Carnitine deficiency affecting transfer of NEFA across the mitochondrial membrane

Patients with FCPD usually require insulin though in small quantities, which is due to the presence of residual beta cell function reflected by the intermediate C- peptide levels seen in these patients [23].

This case had no macrovascular or microvascular complications of diabetes mellitus. Long term microvascular complications of FCPD included; Retinopathy [24] Neuropathy [25], and Nephropathy [26]. In contrast, macrovascular complications were less common [27], perhaps due to the usual young age of onset, their leanness and the low cholesterol levels [27], but has been reported in two case reports of FCPD in elderly [10].

Pain which is a major problem in FCPD, may need surgical interventions if severely intractable and recurrent, evidenced by this patient having relief of pain following annual ERCP and stenting. Surgical options include Drainage procedures, Sphincteroplasty, Pancreatic necrosectomy and Celiac plexus ablation. Early surgery has been found to prevent the development of diabetes in early stages of the disease [28]. The challenges in management are recurrent intractable pain, insulin resistance, recurrent hypoglycemia, malnutrition, inability to adequately correct micro and macronutrient deficiency, poor drug compliance, misdiagnosis and late diagnosis.

## Conclusion

Fibrocalculous Pancreatic Diabetes, though classically described in the young, can occur in the middle aged, without a childhood history of abdominal pain, as shown in this rare case. Therefore a high index of suspicion is necessary in early diagnosis and treatment. Adequate glycemic control with regular monitoring, pain relief, management of macro and micronutrient deficiency and pancreatic failure are important in improving the quality of life in patients with FCPD.

## Consent

Written informed consent was obtained from the patient for publication of this case report and accompanying images. A copy of the written consent is available for review by the editor-in-chief of this journal.

## Abbreviations

FCPD: Fibro calculus pancreatic diabetes
ERCP: Endoscopic retrograde cholangio pancreatography
BMI: Body mass index
US: Ultrasonography
CT: Computed tomography
TSH: Thyroid stimulatimg hormone
CA19-9: Carbohydrate antigen 19-9
IDDM: Insulin dependent diabetes mellitus
NEFA: Non Esterified fatty acids
